# A plant resource and experiment management system based on the Golm Plant Database as a basic tool for omics research

**DOI:** 10.1186/1746-4811-4-11

**Published:** 2008-05-21

**Authors:** Karin I Köhl, Georg Basler, Alexander Lüdemann, Joachim Selbig, Dirk Walther

**Affiliations:** 1 Max-Planck-Institute of Molecular Plant Physiology, Am Mühlenberg 1, 14476 Golm, Germany; 2 University of Potsdam, 14469 Potsdam, Germany

## Abstract

**Background:**

For omics experiments, detailed characterisation of experimental material with respect to its genetic features, its cultivation history and its treatment history is a requirement for analyses by bioinformatics tools and for publication needs. Furthermore, meta-analysis of several experiments in systems biology based approaches make it necessary to store this information in a standardised manner, preferentially in relational databases. In the Golm Plant Database System, we devised a data management system based on a classical Laboratory Information Management System combined with web-based user interfaces for data entry and retrieval to collect this information in an academic environment.

**Results:**

The database system contains modules representing the genetic features of the germplasm, the experimental conditions and the sampling details. In the germplasm module, genetically identical lines of biological material are generated by defined workflows, starting with the import workflow, followed by further workflows like genetic modification (transformation), vegetative or sexual reproduction. The latter workflows link lines and thus create pedigrees. For experiments, plant objects are generated from plant lines and united in so-called cultures, to which the cultivation conditions are linked. Materials and methods for each cultivation step are stored in a separate ACCESS database of the plant cultivation unit. For all cultures and thus every plant object, each cultivation site and the culture's arrival time at a site are logged by a barcode-scanner based system. Thus, for each plant object, all site-related parameters, e.g. automatically logged climate data, are available. These life history data and genetic information for the plant objects are linked to analytical results by the sampling module, which links sample components to plant object identifiers. This workflow uses controlled vocabulary for organs and treatments. Unique names generated by the system and barcode labels facilitate identification and management of the material. Web pages are provided as user interfaces to facilitate maintaining the system in an environment with many desktop computers and a rapidly changing user community. Web based search tools are the basis for joint use of the material by all researchers of the institute.

**Conclusion:**

The Golm Plant Database system, which is based on a relational database, collects the genetic and environmental information on plant material during its production or experimental use at the Max-Planck-Institute of Molecular Plant Physiology. It thus provides information according to the MIAME standard for the component 'Sample' in a highly standardised format. The Plant Database system thus facilitates collaborative work and allows efficient queries in data analysis for systems biology research.

## Background

For omics experiments, a detailed characterisation of experimental material is a critical requirement for a meaningful analysis by bioinformatics tools and for publication needs. The required information primarily depends on the focus of the omics study, e.g. the organ or tissue sampled, the developmental stage, genetic features of the material [1,2], treatment conditions [3] or combinations thereof [4-6]. However, further information is required for a complete description of the material according to the MIAME standards [7,8] or the MSI standards [9]. In addition to information on the experimental treatment, data on the genetic make-up of the experimental material and on the life-history of the organism must be provided. While information directly related to the experimental approach (e.g. the day length and the species in a diurnal cycle experiment) is often recorded in a standardised way together with the results from the analytics in spreadsheet formats, other relevant information, (e.g. the air temperature, the light intensity, the cultivar and the age of the plant) is often restricted to paper based records in lab books or log books of growth facilities and laboriously compiled for publication. Meta-analysis of several experiments in systems biology-based approaches furthermore makes it necessary to store this information in a standardised manner, preferentially in relational databases. Copying this information from traditional paper-based lab-books into databases is time-consuming and prone to errors. Thus, a Laboratory Information Management System (LIMS) that allows collecting this information in an easy and systematic manner during an experiment is an invaluable asset. This is also true for an academic environment, where nowadays several persons are successively involved in the generation of the material, its first characterisation, running omics experiments, data analysis, and – often much later – meta-analysis in systems-biology based approaches. There are a number of challenges in an academic environment. The degree of standardisation in scientific workflows is generally low, as methods are part of scientific progress [10]. Furthermore, the percentage of long-term staff is usually low, which requires intuitive user interfaces. Finally, multi-site projects, in which several academic institutions in different countries are involved, require data management systems that allow efficient data exchange in standardised formats.

Classical LIMS, in which user interfaces are tailored to specific user groups, which are specifically trained to use the system, are thus problematic in an academic environment. In the Golm Plant Database, we solved the problem by combining a classical LIMS with web-based user interfaces for data entry and retrieval. Additionally, on-site data-entry is facilitated by portable barcode-terminals. The LIMS was implemented at the Max-Planck-Institute of Molecular Plant Physiology (MPI-MP), a medium-sized academic institute. In 2006, scientific work was performed by 200 scientists, more than two thirds of which have contracts of less than 40 months. The LIMS implementation was started in the plant growth facility, which is run as a service unit. The climate-controlled and monitored growth area is approximately 1,400 m^2^, in which about 170,000 experimental plant objects were grown in 2006. The system was set up since 2002 and introduced stepwise to all users between December 2004 and February 2006. It replaced an older system, in which information on plasmids was stored in an ACCESS database and information on production of transgenic plants and greenhouse workflows was recorded on paper form sheets. Here, we present the design of the plant database system with its user interfaces and the results of the utilization of the system during a period of two years.

## Results

### Germplasm and plant objects

The genetic diversity of plant material that is used in research at the institute is represented by plant lines. A plant line comprises a batch of plants that can be regarded as one genetic entity. For example, all seeds in an imported seed package, or all seeds derived from a certain crossing, or the material generated in a transformation in progress would each be represented by a plant line. When plants from a line are used in an experiment or grown for propagation, so-called plant objects are generated. Plant objects represent one or several plants regarded as one entity in an experiment.

**Figure 1:**
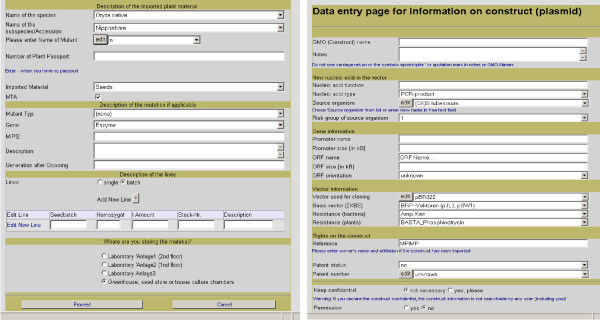
Web pages for the import of plant lines (left) and additional form for the description of constructs used to generate (imported) transgenic plants (right).

The information on plant lines is entered into the system when the material is either imported into the institute or produced in the institute. The data entry forms are available as web pages that require personal login, thereby recording the ownership of the material. The import process is the basic process. In this process, information on the origin of the material and its properties are recorded (Fig. 1). Some of the information can be chosen from drop-down menus containing controlled vocabulary (e.g. name of the species, name of the subspecies or accession). Other fields are drop-down menus (e.g. name of mutant), to which names can be added by the user. Description fields, e.g., allow free-text entry. In case of transgenic plants, construct data are entered on a separate form (Fig. 1). Here, legally required information, e.g. on the function of the introduced gene and its source organism, are requested.

**Figure 2:**
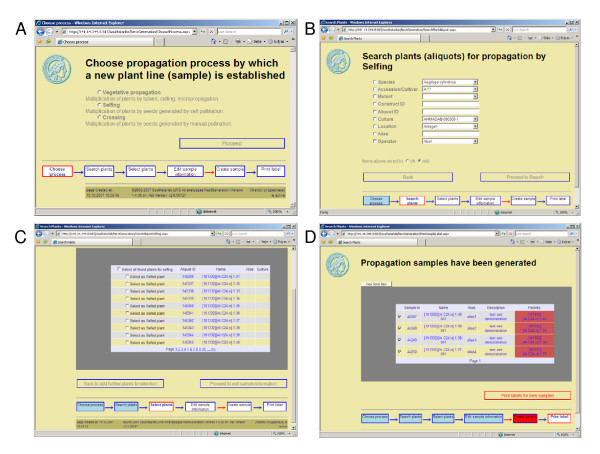
Web pages for propagation. A) Selection of a propagation process. B) Search tool for plant objects. C) Selection page. D) Results page with label printing function. Flow diagrams at the bottom stage indicate the subsequent data entry step: a red frame indicates the active step, blue or red background filling indicate done steps, red arrows indicate committing steps that trigger data transfer to the database.

Internal production processes like plant transformation, vegetative or generative propagation, or crossing are based on plant objects from existing plant lines, which have been either directly imported or generated in a previous production process. The scientist first chooses the process, e.g. the type of propagation (Fig. 2A) or a transformation technique. For propagations, the available parents can then be searched by different criteria in the list of available plant objects (Fig. 2B) and picked from the results list (Fig. 2C). For transformation, plant information needs to be linked to the construct information. The researcher can enter data on new constructs on the same form that is used for the import (Fig. 1) or select from a list of existing constructs. Then, the transformed plant can either be chosen from a list of standard parents or searched again in the list of available plant objects by several criteria. All information (source, species, and subspecies) that was entered before for the plant line, from which the parent object is derived, is thus available for the newly generated plant line and does not need to be entered again.

When the data entry is completed, data are submitted to the database and automatically linked to the name of the person who logged on to the web page. The researcher receives a feedback with unique names and identifier numbers of the newly generated plant lines (Fig. 2D).

As a result of these workflows, data on 16,191 plant lines have been entered into the system by 342 researchers, 134 of which have already left the institute (survey on 9 March 2008). This dataset includes legacy data from the time before the introduction of the system. However, even when the survey is restricted to the last two years, during which the system was accessible to all researchers and in which 12,200 lines have been entered, 35 out of 205 researchers had already left at the time of the survey. This indicates how rapidly the material changes hands and how important a systematic data storage is. Among all lines described in the system, 26% have been imported to the institute, 20% produced by transformation processes and the remaining 54% originate from propagation and crossing procedures. Thus, for almost 75% of the material, the institute is the primary source for the germplasm and the information on it.

All plant lines in the germplasm module of the system are searchable by different parameters on search pages. Group specific pages for the search results display the genetic resources of each research group. On these pages, entries into description fields and alias names (given names without unique constraints) can be made and edited by group members. Additionally, standardised labels with name and identifier in clear text and barcode can be printed for each plant line of the research group. Result pages showing all lines of the institute display the genetic information, the origin of all germplasms and the name of the research group that owns the material.

### Cultivation experiments

**Figure 3:**
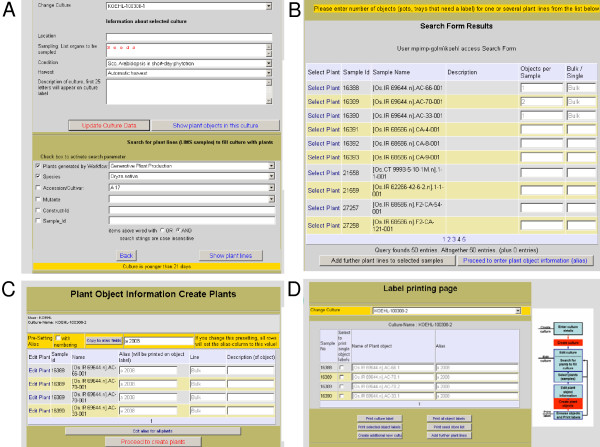
Web pages for plant cultivation experiments. A) Information on culture (upper half) and search tool for plant lines. Location displays the actual location when the culture is in the greenhouse. The search tool is used to add plant objects from existing plant lines to the culture. B) Search result screen with three lines, for which the generation of plant objects has been selected. C) Editing screen, on which alias names and descriptions for each object can be entered, before the selection is submitted. D) Result screen showing all objects in a culture with print functions for labels and reports, and buttons that link back to the search page (A) or to a page, on which new cultures can be generated.

In cultivation experiments or propagation procedures, single plants or groups of plants that are regarded as one entity are represented as plant objects. An object can thus be one plant, but also a group of plants grown in the same container, or a population of cells growing in the same vessel. On the plant cultivation web page, the researcher enters the unique identifier of a plant line from the seed vial or searches the plant line in the germplasm module (Fig. 3A). On the result page, the numbers of plant objects to be grown per plant line are entered (Fig. 3B). Then, user selected alias names and descriptions can be entered for each object (Fig. 3C). After submission, uniquely labelled and numbered plant objects are generated and displayed on a response screen (Fig. 3D). To facilitate handling, all plant objects that are planned to undergo the same cultivation process are grouped together in a so-called '*culture*'. Information about the responsible scientist and his affiliation, the start date of the experiment and the cultivation protocol are attached to this *culture*. The cultivation protocol is defined based on a list of pre-defined standard cultivation protocols that describe every step of the cultivation procedures including the materials (vessels, substrates, fertilizers) used. Standard cultivation protocols are documented outside of the LIMS to allow different documentation formats. For highly standardised processes in service units, protocols are stored in a separate database hosted by the service unit [see additional file 2]. Text documents are preferred for more rapidly changing, less standardised processes in scientific research groups.

For each culture and each object in a culture, an automatically generated label is produced and its printing trigged on the web page (Fig. 3D). Object labels display the name and the alias of the object; additionally, the object identifier is printed as clear text and barcode. The *culture *labels show the name of the culture in clear text and in barcode. This barcode is used to monitor the location history of each culture in the cultivation facilities. Upon arrival at a cultivation site, the barcode of the culture name and the barcode label of the cultivation area are scanned with a portable barcode-scanner data terminal. Data are automatically transmitted to the LIMS when the data-terminal is placed in its cradle; the entry receives the time stamp of the login. Thus, no extra data entry step on the computer keyboard is required to document processes in the plant cultivation area. This facilitates the integration of the data management into the work-processes on the greenhouse sites. The process is repeated whenever the location of the culture is changed. Thus, the entire location history for each culture and, consequently, of each plant object in a culture can be reconstructed. The knowledge of the location provides access to information that is stored for each location, namely plant protection measures, climate settings and climate records for each climate controlled area.

Within two years (3 February 2006 to 2 February 2008), these data were recorded for 6,248 cultivation experiments performed by 260 researchers, 60 of which had left the institute before the 5 March 2008. Among the approximately 6,000 plant lines that have been used by the researchers for cultivation experiments, 143 have been used by five or more researchers, 58 by five or more different research groups.

### Sampling

**Figure 4:**
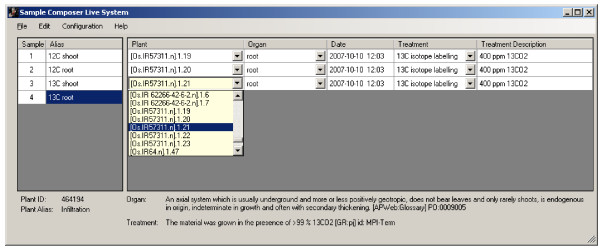
In the Sample Composer, a component of a sample is defined by selecting one plant object from the filtered object list and combining it with information about sampling time, organ and treatment information. Definitions of the ontology terms, identifier and alias of the selected plant object are displayed at the bottom of the page. The example shows four samples, the fourth sample has three components.

The process of sampling from a plant is the final step to link the information about a plant's genetic make-up and the environmental conditions, to which the plants were exposed, to the data that were derived from tests performed on the plant material, especially metabolic profiling. These tests can be performed on material that is generated by pooling material taken from more than one plant object. Therefore, each sample is composed of one to many sample components that link to single plant objects. For each component, the user interface Sample Composer (Fig. 4) records the plant object, from which the material is taken, the sampling time, the sampled organ and the treatment. Organ and treatment class are selected from controlled vocabulary lists [11]. The published organ and treatment ontologies [12] were complemented by additional terms. Each organ and treatment term is supplemented by a definition, which is displayed in the Sample Composer. An additional treatment description allows specifying any deviation from the above-mentioned standard cultivation condition. The unique sample identifier and an automatically generated, unique name provide the link between the test results, e.g. metabolic or expression profiles and the data on the plant and its life-history.

## Discussion

We set up a LIMS for the plant cultivation facility at the MPI-MP with the aim to provide efficient access to genetic and environmental data on all plant materials for optimal data mining as a basis for systems biology. Systems biology approaches require large data sets in which results data (dependent variables) from metabolic or transcript profiling approaches are combined with metadata (independent variables) about the genetic make-up and the environmental parameters of the experimental material, on which the measurements were performed. Typical research projects in the systems biology field involve many researchers from several research groups with biological or technical focus (e.g. metabolomics, proteomics). The biological material used in the project is often produced and characterised by other researchers or imported from external sources. Although our survey was limited to a two year period, the personnel working on the material changed considerably during that period. Furthermore, a considerable number of plant lines were used by more than 25% of the experimentally-working research groups. Before the introduction of the system, the required metadata were generally documented in different formats across many sites with a very low degree of standardisation. This meant that a direct data transfer to results databases, e.g. the Golm Metabolome Database [13], had to be done manually and, thus, was inefficient and prone to errors and data loss. We thus decided to build a standardised, centralised database system to collect this meta-information on-site and at the time when the processes, e.g. import of plants, generation of new genetically modified organisms, planting of plants or sampling of biological material, take place. One advantage of a centralised system is the option to share information, thus avoiding double entry of data when several researches from different groups are using the same material. On the other hand, seemingly identical material derived from different sources or different propagation steps are treated as separate plant lines, so that meta-analysis can analyse the effect of the source or propagation step on the result and thus detect seed batches that yield aberrant patterns.

The second advantage is the common data format for all information that is the basis for automatic queries. These queries are, for example, used on web pages that allow searching the collection for plant lines with specific features. The information, whether material of the required specification is in the institute and who has been working on it, facilitates communication and the exchange of material. The LIMS thus helps to make maximum use of the large and both scientifically and financially precious germplasm collection of the institute. Queries to the Golm Plant Database can also be part of data mining tools, in which data from the Golm Plant Database are combined with results data from other databases like the Golm Metabolome Database for metabolites [13], the ProMEX database for proteins and their phosphorylation sites [14], the NASCArray data base for microarray data [15] or other LIMS like the Genotyping LIMS of the ICRISAT [10].

There are a number of challenges in the implementation of a central LIMS in a medium-sized research institute. One challenge is the import of legacy data from sources with a low degree of standardisation, frequently from paper documents. When the system was started, information about existing plant material had to be entered into the system. This is a labour intensive step that has to be performed by people with expert knowledge about the material.

The second challenge is the low degree of standardisation in research in comparison to the typical service laboratory, for which LIMS are generally designed. We approached this problem by defining standard protocols for those processes in service units, for which a high degree of standardisation is achieved. For the individual experiment, the researcher can select the protocol from a list and modify it by additional comments in description fields attached to the culture or the plant object. A certain degree of standardisation in these descriptions was achieved by using predefined forms, e.g. for the description of a sample, that use controlled vocabulary instead of free text entries for the first classification of a sample.

The third, and perhaps biggest, challenge is the training and support of the system users. Even a very simple user interface is a big difference to the accustomed free-text documentation. The user interface requires information in a more standardised and often also more complete format than documentation systems based on free-text lab-books. The time between the extra effort and the advantage gained is generally long, and often the person who has to enter the data and the person reaping the benefit are not identical. The initial motivation of an individual researcher to familiarize himself with a LIMS is thus not very high. The start of a LIMS therefore needs a sufficiently high number of people involved in individual training and support of the users. The problem can be partially solved by splitting the start phase into several modules so that subgroups rather than the entire scientific staff of an institute are introduced to the system. After the setup and introduction phase, the system needs further maintenance, both on the side of database administration and maintenance of controlled vocabulary as well as on the side of user support by tutorials and the implementation of workflows for new techniques.

The plant database system that has been operated at the MPI-MP in Golm provides most of the information required by MIAME/Plant for the MIAME component 'Sample'. For each sample generated by the Sample Composer, the link between the (pooled) sample components and the plant object, which is itself linked to the plant line, provides access to many of the biosource properties and biomaterial manipulations data required by MIAME/Plant. The plant line dataset directly, or by pedigree search, provides information on the taxonomic species name, the subspecies or cultivar, mutations and, if applicable, genetic modifications by transformation techniques. Furthermore, the information about the generation process (crossing, generative or vegetative propagation) delivers information about the material (seeds or cuttings) and – for material produced at the MPI-MP – the age of the starting material. The link between the plant object, generated from the plant line, and the culture provides access to information linked to standard procedures, namely the substrate, vessels, and fertilizers that are used in each step, and the environmental conditions, under which the material is to be grown in each step. These protocols include information on standard treatments (e.g. seed treatments after harvest and before sowing) and vernalisation procedures. Furthermore, the link between culture and location, which allows following the location history of each plant object, provides information about the real climate conditions during the cultivation and about unplanned events, like pesticide treatments. The information about the part of the organism sampled for an experiment is provided when the sample component is generated by the Sample Composer. The difference between sampling date and the time when the plant culture was first scanned to a cultivation site provides the age of the plant. The sampling times give access to the climate conditions (temperature, humidity and light intensity) during and before sampling, including information on the sampling time with respect to the diurnal cycle. Thus, many parameters relevant for expression or metabolic profiling are automatically recorded in a highly standardised format. Treatments specific to an experiment, however, are entered in a free-text description field and are thus not standardised. A first classification is possible by selecting terms from the treatment classification list.

## Conclusion

The Golm Plant Database system was implemented based on a LIMS enhanced with web interfaces in a medium-sized academic institute. Data on the generation of plant material and cultivation experiments with the materials are collected during these processes and stored in a relational database. Thus, for plant derived samples, the system provides most of the information, both on genetic as well as environmental features, required for the MIAME component 'Sample' in a highly standardised format. Furthermore, the central information repository about germplasm of the institute facilitates collaborative use by different research groups and for longer time spans. Automatically generated barcode labels with unique names furthermore reduce the risk of mix-up and facilitate storage management. We are thus confident that the system makes work at the institute more efficient and generates reliable datasets suitable for meta-analysis in omics and systems biology projects.

## Methods

### Software and Hardware

The Golm Plant Database is implemented within the commercial LIMS Nautilus 2003 R2 B3 (ThermoFisher, Dreieich, Germany). The LIMS is based on Oracle9i (Oracle Corporation, Redwood City, USA). The live system is presently operating under Windows 2000 Server (Microsoft Corporation, Redmond, USA). The test system is operating under Windows Server 2003 (Microsoft Corporation). Both systems are operating on a Dell PowerEdge 1950 system (Dell, Round Rock, USA).

User interfaces were programmed as ASP.NET 1.1 and 2.0 (Microsoft Corporation) web pages for Internet Information Server 5.1 and 6.0 (Microsoft Corporation). The web pages and the sampling module generate XML-Files that are forwarded to the LIMS' background processor by the Thermo-event monitor program (ThermoFisher). Barcode-scanner based data entry is performed with the portable data terminals CipherLAB 711-L (AISCI, Bad Salzuflen, Germany). Data transfer from the scanner to the server is performed by the program 232_read.exe (AISCI). CSV Files from the data terminals are converted into XML files by a PERL-program (ActivePerl 5.8.0 Build 806, ActiveState, Vancouver, Canada).

The sampling module was programmed with Visual Studio 2005 and C# (Microsoft Corporation), and communicates with Oracle 9i using Oracle Instant Client 10.1.0.5 (Oracle Corporation). Data from the ontologies and plant objects from the LIMS are cached to speed up local data operations, before performing the XML import and response file generation.

The LIMS objects layout is given in the additional file 1.

The ACCESS-Database of the service unit is implemented in ACCESS 2000 (Microsoft Corporation). The data model for the relevant table is given in the additional file 2.

### Germplasm module

The LIMS concept of a sample is used for any plant line, which means any batch of plants (seeds, tubers, whole plants) derived from the same workflow step [see additional file 1]. So far, the workflows 'import of plants', 'transformation of plants', 'vegetative' and 'generative propagation' and 'crossing' have been implemented. Any individual plant object from that batch is represented by the LIMS concept of an aliquot. A plant object is defined as one or several plants that are regarded as one object in an experiment. Each plant object is linked to a plant line by entering the identifier number of the parent plant line into the object record.

In the starting workflow 'import of plants', the key information 'species' (including 'subspecies'), 'cultivar' or 'ecotype' and known 'mutations' of the imported material are entered. Furthermore, information about the supplier, the import date and the person and research group by which the material was imported is stored. For genetically modified organisms (GMO), the legally required information for the complete description of the plasmid plus additional information necessary for the efficient use of the plasmid (e.g. antibiotics resistance markers) are entered on a separate construct-information page. The information is added to a construct-information table and the construct dataset is flagged as 'imported'. After data submission, the newly generated plant line receives a unique identifier number and an automatically generated unique name. The name is generated from the species abbreviation, cultivar and mutant name. In case of genetically modified material, the primary key of the construct information table is added as a prefix to the name. An additional counter at the end of the name allows to automatically generating unique names for each plant line, even if the same material is imported several times or from several sources.

In the workflow 'transformation', a new line of genetically modified plants is generated by linking a parent plant object to construct information. The parent relation is represented as a link from the newly generated plant line to the parent plant object that was used in the transformation. The construct information is given as a link to the microorganism line that was either used to perform the transformation (in case of an Agrobacteria mediated transformation) or to generate the DNA used in transformation (in case of a ballistic transformation). The dataset of each microorganism line contains a link to the construct, with which the microorganism has been transformed, and additional information on the species and strain. The name of the new plant line produced by transformation is generated from the primary key of the construct data set and the name of the transformed parent plant object.

For the workflows 'generative propagation' that models the self-pollination of a plant, and 'vegetative propagation', the parent relation is represented as a link to one plant object. The name of the newly generated line is derived from the name of this plant object, thus containing the plant object number of the mother plant. Different separator symbols in the syntax (dot or minus) allow to distinguish between vegetatively and generatively produced plant lines. 'Crossing' models the process where pollen-acceptor and -donor are different plants. The links to the primary keys of the respective plant objects are stored in two object (aliquot) link fields of the newly generated plant line. The name of the newly generated line is derived from both parent plants. An additional counter in the name allows generating unique names for individual lines that are produced by the independent repetition of a workflow. In addition to the automatically generated unique name and number, a description field and a so-called 'alias' field allow the user to enter further information on any line and object, and to give it a name of his choice that is not under unique constraint.

When plant objects, represented as aliquots, are generated from the line, the name of the object is generated from line name plus a counter. Thus, a unique name is generated, which contains information on the pedigree of the plant object.

### Plant cultivation module

To facilitate handling, every cultivated plant object belongs to a so-called '*culture*', represented by the LIMS object 'study' [see additional file 1]. The primary key of the culture is added to the plant object record. Each culture is uniquely named based on the truncated name of the person who entered the *culture *into the database, the entry date and a counter. Information about the responsible scientist and his affiliation and about the cultivation protocol is attached to each *culture*. The cultivation protocol is selected from a list of pre-defined standard cultivation protocols. For highly standardised processes in service units, protocols are stored in an MS-ACCESS database. For each step of the protocol, the relative time (days after start), the method description and the media (vessels, substrate, fertilizer, nutrient solutions, treatment solutions) and the climate conditions are recorded [See additional file 2].

For each culture and object in a culture, an automatically generated label is produced. The labels are defined in SYBASE INFOMAKER (Sybase) and produced by LIMS reporting workflows. The printing is triggered by submitting an XML file to the LIMS background processor. Object labels display the name, alias and identifier of the object as clear text; additionally, the identifier is displayed as barcode. The *culture *labels show the name of the culture in clear text and in barcode, the research group of the owner and the first 25 letters of the description field.

In the greenhouse, the barcode of the culture name and the barcode label of the cultivation area are scanned with a portable barcode-scanner data terminal. Data are automatically transmitted to the LIMS when the data-terminal is placed in its cradle; the entry receives the time stamp of the login. For data transfer, CSV (comma separated values) data from the barcode scanner are converted into XML (Extensible Markup Language) files by a PERL script. This step allows running several different workflows, which yield different data formats, on the same scanner. Each output line is converted into the XML format required by the LIMS import interface. For each movement of plant material, the *culture *name, the name of the old location, the name of the new location and the date are stored in an audit table of the LIMS.

Cultivation sites are organised hierarchically to allow inheritance of information from a higher-order unit to a lower-order unit, e.g. from a greenhouse to an individual greenhouse cabin to an individual bench in a cabin. For each cabin or bench, plant protection measures and the climate settings are stored in the MS-ACCESS database of the greenhouse service unit. For each plant protection measure, treated cultivation sites, the date and time, the size of the treated area, the pesticide, its volume and its concentration are stored. Climate records comprise begin and end of the artificial light day, the temperature and the humidity in both light phases. For the greenhouse, additional settings are stored, e.g. alarm temperature thresholds and light intensity thresholds for automatic closure of the shading system. The light intensity, temperature and humidity records from the sensors in the cabins are automatically collected every 20 minutes and stored in the database of the climate control system.

### Sampling module

The Sample Composer displays the available plant objects from preselected *cultures*. For each component, the user enters the plant object, the sampling time, the sampled organ, the treatment class and an additional free-text treatment description [see additional file 1]. After submission, unique sample identifiers and names are generated automatically by the LIMS and exported together with sampling information and information about the sampled plants (pedigree, location, cultivation conditions) to EXCEL compatible, tab-separated files.

### Availability and requirements

The ACCESS database, PERL scripts and XML files for data transfer, the scanner programs and screenshots of all web pages are freely available to non-commercial users from the corresponding author. Source code and demo version of the Sample Composer are included in additional file 3. The LIMS part of the system is based on a commercial LIMS and an Oracle database (see Methods, Software and Hardware). The ER diagram and the description of those tables and variables used in the system that has been described in the manuscript are available from the corresponding author and can be used as a basis for the implementation of a similar system in a relational database.

Project name: Golm Plant Database. Project home page: . Operating systems: Windows 2000 or Windows 2003. Programming language and other requirements: see Methods, Software and Hardware.

## List of abbreviations

LIMS: Laboratory Information Management System; MIAME: Minimum information about a microarray experiment; PERL: Practical extraction and report language; XML: extensible markup language.

## Competing interests

The authors declare that they have no competing interests.

## Authors' contributions

KK designed the architecture for the germplasm and cultivation modules and the web based user interfaces, performed the import of the legacy data, programmed the portable scanners and evaluated the system. The implementation work for the germplasm module and the cultivation module in the LIMS and the programming of the web pages was done by external programmers (Thermofisher, Southstarlab). GB programmed the LIMS sampling workflows and the Sample Composer. AL programmed the PERL-script. DW and JS initiated and supervised the work of GB. All authors read and approved the final manuscript.

## Supplementary Material

Additional file 2 Access. Diagram displaying the entity-relation-model of the ACCESS database used by the plant cultivation service. Upper half displays tables to describe standard cultivation protocols and their link to the LIMS. In this part, the cultivation steps and the containers, substrates, fertilizers and nutrient solutions used in these steps are documented. The lower half shows tables containing information on plant cultivation sites (locations), namely on the reservation status and on the climate conditions between defined dates and on pesticides that have been applied in these locations on a defined date. Objects are depicted as rectangles containing the respective attributes, foreign key relations as arrows. Primary key attributes are underlined. Click here for file

Additional file 1 NautilusOracle. LIMS objects representing plants, plant lines, cultures and samples. Objects are depicted as rectangles containing the respective attributes, foreign key relations as arrows. Primary key attributes are underlined and denoted by PK, foreign keys by FK1 to FK10. Mandatory attributes are printed bold. Click here for file

Additional file 3 Samplecomposer. The zip-file contains the source code and a demo version of the sample composer and the controlled vocabulary for organ and treatment as csv files. Click here for file
