# Supplementary material for: A plant resource and experiment management system based on the Golm Plant Database as a basic tool for omics research
Source: Plant Methods. 2008 May 21;4:11. doi: 10.1186/1746-4811-4-11 (PMC2409336; doi:10.1186/1746-4811-4-11)
Supplement: Additional file 2 — Access. Diagram displaying the entity-relation-model of the ACCESS database used by the plant cultivation service. Upper half displays tables to describe standard cultivation protocols and their link to the LIMS. In this part, the cultivation steps and the containers, substrates, fertilizers and nutrient solutions used in these steps are documented. The lower half shows tables containing information on plant cultivation sites (locations), namely on the reservation status and on the climate conditions between defined dates and on pesticides that have been applied in these locations on a defined date. Objects are depicted as rectangles containing the respective attributes, foreign key relations as arrows. Primary key attributes are underlined. [file 1746-4811-4-11-S2.pdf]

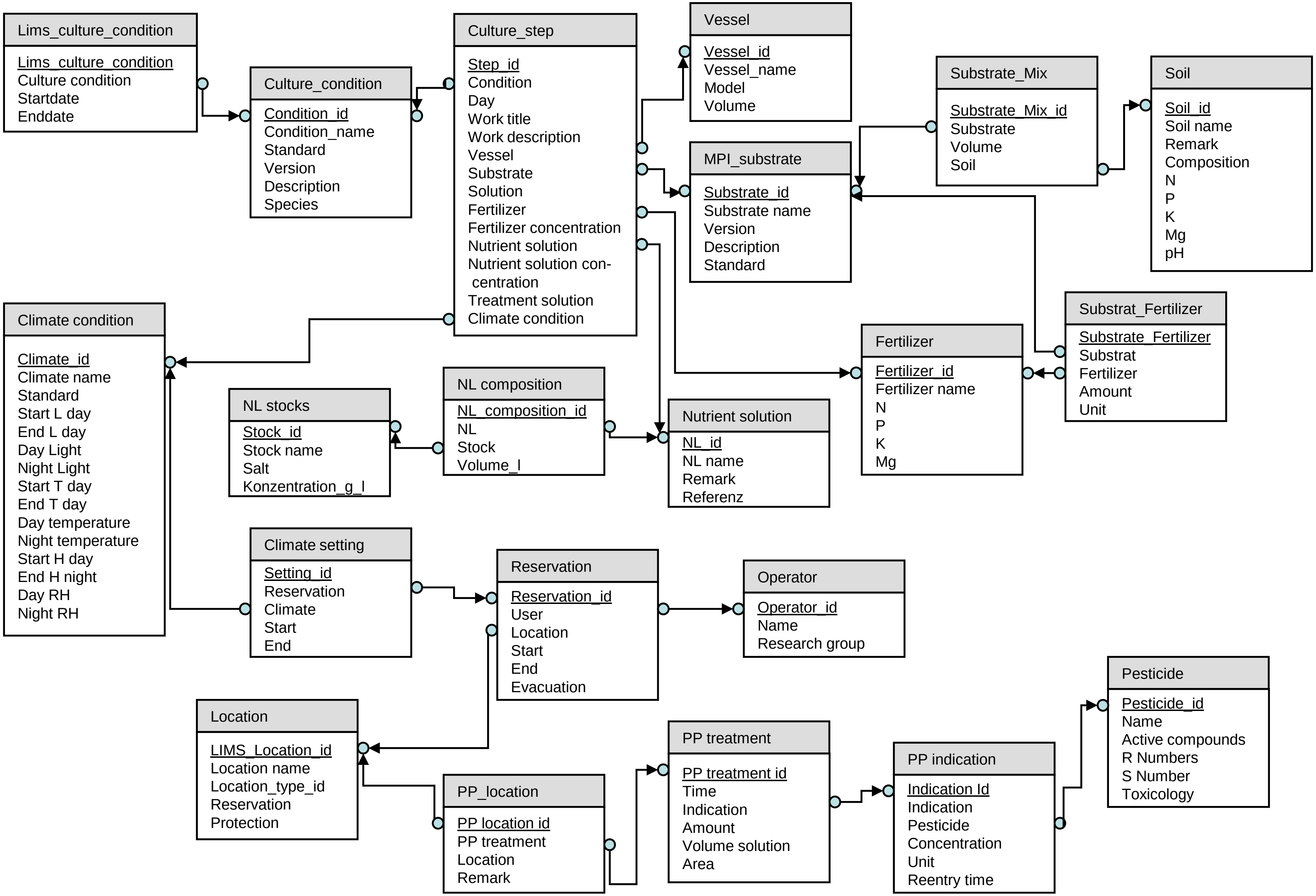

Diagram displaying the entity-relation-model of the ACCESS database used by the plant cultivation service. Upper half displays tables to describe standard cultivation protocols and their link to the LIMS system. In this part, the cultivation steps and the containers, substrates, fertilizers and nutrient solutions used in these steps are documented. The lower half shows tables containing information on locations like reservation status and climate conditions in plant cultivation sites (locations) and pesticides that have been applied in these locations. Object are depicted as rectangles containing the respective attributes, foreign key relations as arrows. Primary key attributes are underlined.
