# Supplementary material for: A plant resource and experiment management system based on the Golm Plant Database as a basic tool for omics research
Source: Plant Methods. 2008 May 21;4:11. doi: 10.1186/1746-4811-4-11 (PMC2409336; doi:10.1186/1746-4811-4-11)
Supplement: Additional file 1 — NautilusOracle. LIMS objects representing plants, plant lines, cultures and samples. Objects are depicted as rectangles containing the respective attributes, foreign key relations as arrows. Primary key attributes are underlined and denoted by PK, foreign keys by FK1 to FK10. Mandatory attributes are printed bold. [file 1746-4811-4-11-S1.pdf]

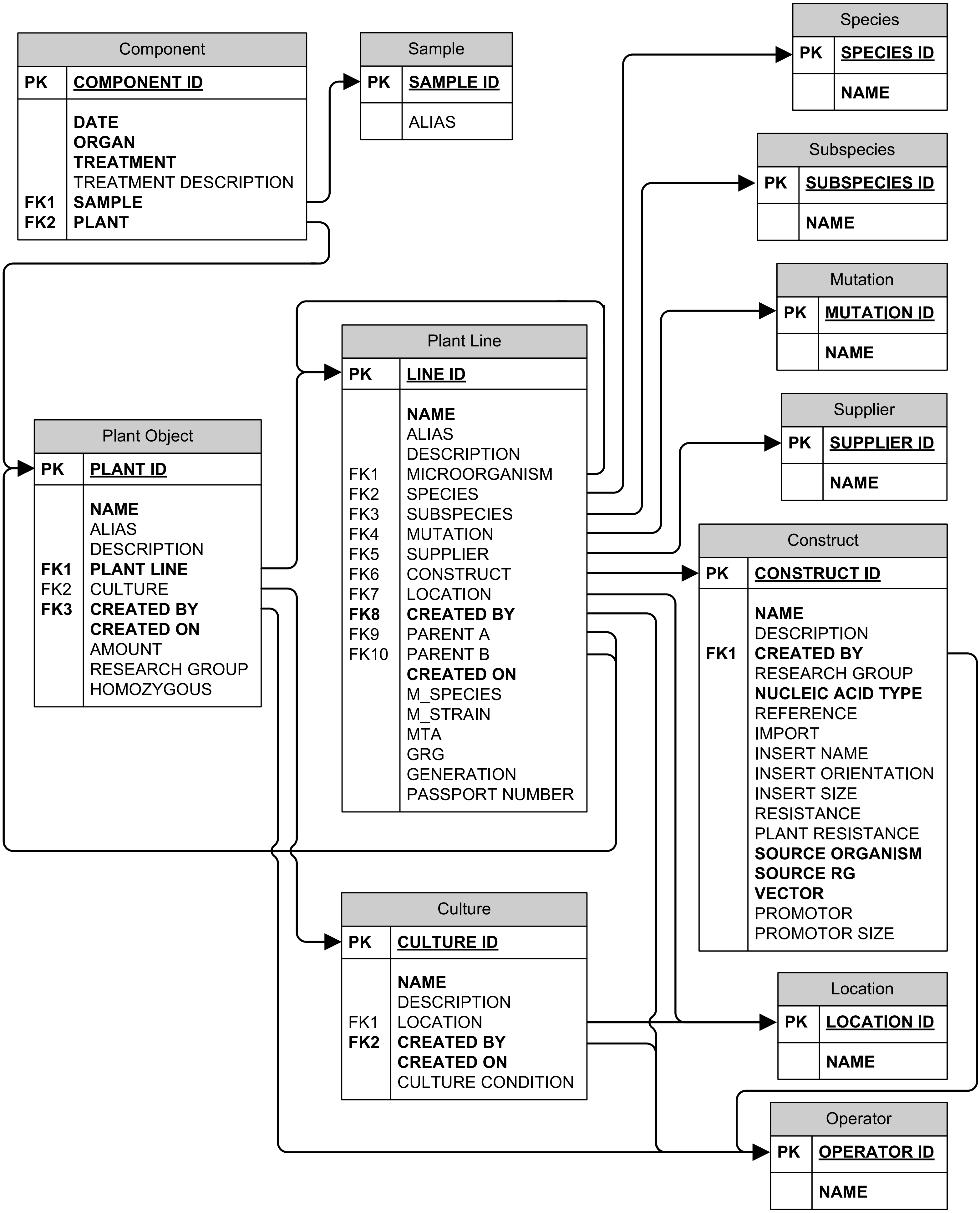

LIMS objects representing plants, plant lines, cultures and samples. Objects are depicted as rectangles containing the respective attributes, foreign key relations as arrows. Primary key attributes are underlined and denoted by PK, foreign keys by FK1 to FK10. Mandatory attributes are bold.
